# Review of Materia Medica for the Use of Students

**Published:** 1852-04

**Authors:** 


*Review of Materia Medica for the use of Students.* By John Biddle, M. D., &c. &c. With illustrations. Philadelphia: Lindsay & Blakiston, 1852, pp. 320.

A very convenient and good book for students and physicians. Dr. Biddle is an excellent writer, and has richly earned, and will receive the thanks of his medical brethren for the valuable work which he has presented to the profession.

